# Anemonefish facilitate bleaching recovery in a host sea anemone

**DOI:** 10.1038/s41598-020-75585-6

**Published:** 2020-10-29

**Authors:** Sophie H. Pryor, Ross Hill, Danielle L. Dixson, Nicola J. Fraser, Brendan P. Kelaher, Anna Scott

**Affiliations:** 1 National Marine Science Centre, Marine Ecology Research Centre, School of Environment, Science and Engineering, Southern Cross University, P.O. Box 4321, Coffs Harbour, NSW 2450 Australia; 2 Macquarie University, Sydney, NSW 2109 Australia; 3 School of Marine Science and Policy, University of Delaware, Lewes, DE 19958 USA

**Keywords:** Ecology, Microbiology

## Abstract

Ocean warming is causing the symbioses between cnidarians and their algal symbionts to breakdown more frequently, resulting in bleaching. For sea anemones, nutritional benefits derived from hosting anemonefishes increase their algal symbiont density. The sea anemone-anemonefish relationship could, therefore, facilitate bleaching recovery. To test this, bleached and unbleached sea anemones, both with and without anemonefish, were monitored in the laboratory. At the start of our experiment, algal symbiont density and colour score were lower in the bleached than unbleached sea anemones, whereas total chlorophyll remained similar. After 106 days, bleached sea anemones with anemonefish had an algal symbiont density and colour score equal to the controls (unbleached sea anemones and without anemonefish), indicating recovery had occurred. Furthermore, total chlorophyll was 66% higher in the bleached sea anemones with anemonefish than the controls. In contrast, recovery did not occur for the bleached sea anemones without anemonefish as they had 78% fewer algal symbionts than the controls, and colour score remained lower. Unbleached sea anemones with anemonefish also showed positive changes in algal symbiont density and total chlorophyll, which increased by 103% and 264%, respectively. Consequently, anemonefishes give their host sea anemones a distinct ecological advantage by enhancing resilience to bleaching, highlighting the benefits of symbioses in a changing climate.

## Introduction

Symbioses, where dissimilar organisms have evolved to coexist, are essential for maintaining ecosystem functions^1–3^. These associations can alleviate climate change impacts^4–6^. For instance, the relationship between endophytes and rice reduces host water requirements and facilitates drought tolerance^7^; and coral reef Trapeziid crabs clean sediments from habitat-providing corals, supporting host survival and growth^8^. Anthropogenically induced stressors of natural ecosystems are increasing globally, and furthering our knowledge of symbioses may offer new insights into mechanisms that enhance organisms' resilience to threats such as climate change^9^.

The symbioses between habitat-forming cnidarians, such as corals and sea anemones, and unicellular algae of the family Symbiodiniaceae provide a major source of primary production in many marine systems^10^. These algae are located within the cnidarians' gastrodermal tissue, where they photosynthesise producing sugars that support host growth and reproduction, and positively influence host fitness^11–13^. Here, the endosymbionts gain access to in hospite nutrients, such as ammonium, improving their condition and density^13–17^. However, during times of environmental stress, this relationship can breakdown, with the loss of Symbiodiniaceae and or their photosynthetic pigment (chlorophyll) causing the host to lighten and become ‘bleached’^18^. Bleaching reduces the photosynthates available to the host, and if the cnidarian remains bleached for a prolonged period, starvation can result in mortality^19,20^. Consequent ecosystem-wide changes can occur, such as decreased coral and sea anemone cover^21–23^, algal dominated phase shifts^24–26^ and long-term decreases in fish abundance and biodiversity^27–29^.

All ten sea anemone species that provide habitat for obligate symbiotic anemonefishes are susceptible to bleaching^30^. Bleaching has been documented in numerous tropical and subtropical locations throughout their Indo-Pacific distribution and can result in sea anemone mortality^31–34^. Subsequent reductions in anemonefish abundance occur as anemonefishes cannot survive in the field without their host sea anemones^31–34^. Depending on the severity, sublethal impacts can occur to the sea anemones, such as oxidative stress and decreased size^33, 35^. Bleached sea anemone habitat has cascading consequences for anemonefishes, including decreased fecundity, increased metabolic demand, increased stress hormones, and vulnerability to predators due to behavioural changes^36–38^.

Anemonefishes provide numerous benefits to their host sea anemones, including excreting metabolic waste in the form of ammonia^17^. Ammonia bonds to hydrogen ions in seawater, becoming ammonium. As a result of the anemonefishes' close physical proximity to their host sea anemone, the ammonium assimilates in the sea anemone cytoplasm via inward diffusion^14,15^. Symbiodiniaceae then absorb ammonium via reverse translocation^39^. Through this nutritional pathway, anemonefish presence increases endosymbiont density^17^. As Symbiodiniaceae density increase, so too do the photosynthates available to the sea anemone, and therefore when sea anemones are occupied by anemonefish, they have higher growth, asexual reproduction, tentacle expansion, and survival^40–42^.

Given the nutritional benefits of hosting anemonefishes, we aimed to determine if anemonefish can facilitate Symbiodiniaceae recovery in a host sea anemone following thermal bleaching. *Entacmaea quadricolor*, the bulb-tentacle sea anemone*,* was used as it is geographically widespread, relatively abundant, and provides habitat for 13 anemonefish species^43^. Furthermore, *E. quadricolor* is vulnerable to ocean warming, having a bleaching threshold of ~ 1 °C above the current summer maximum in subtropical eastern Australia^44^. Bleached and unbleached sea anemones were assigned to the following treatments: with an adult anemonefish pair (*Amphiprion akindynos*); without anemonefish (control) and; without anemonefish but with inaccessible fish food pellets added to the aquaria (procedural control). Recovery was deemed to have occurred when the response variables in the bleached sea anemones were statistically similar to the unbleached controls. We hypothesised that: (i) bleached sea anemones with anemonefish would recover, with no significant difference in Symbiodiniaceae density, total chlorophyll, or colour score in comparison to unbleached control sea anemones; (ii) bleached sea anemones without anemonefish would be unable to recover during the experimental timeframe; and (iii) unbleached sea anemones with anemonefish would have increased Symbiodiniaceae density and total chlorophyll due to the nutritional benefits received.

## Results

### Symbiodiniaceae density

**Figure 1:**
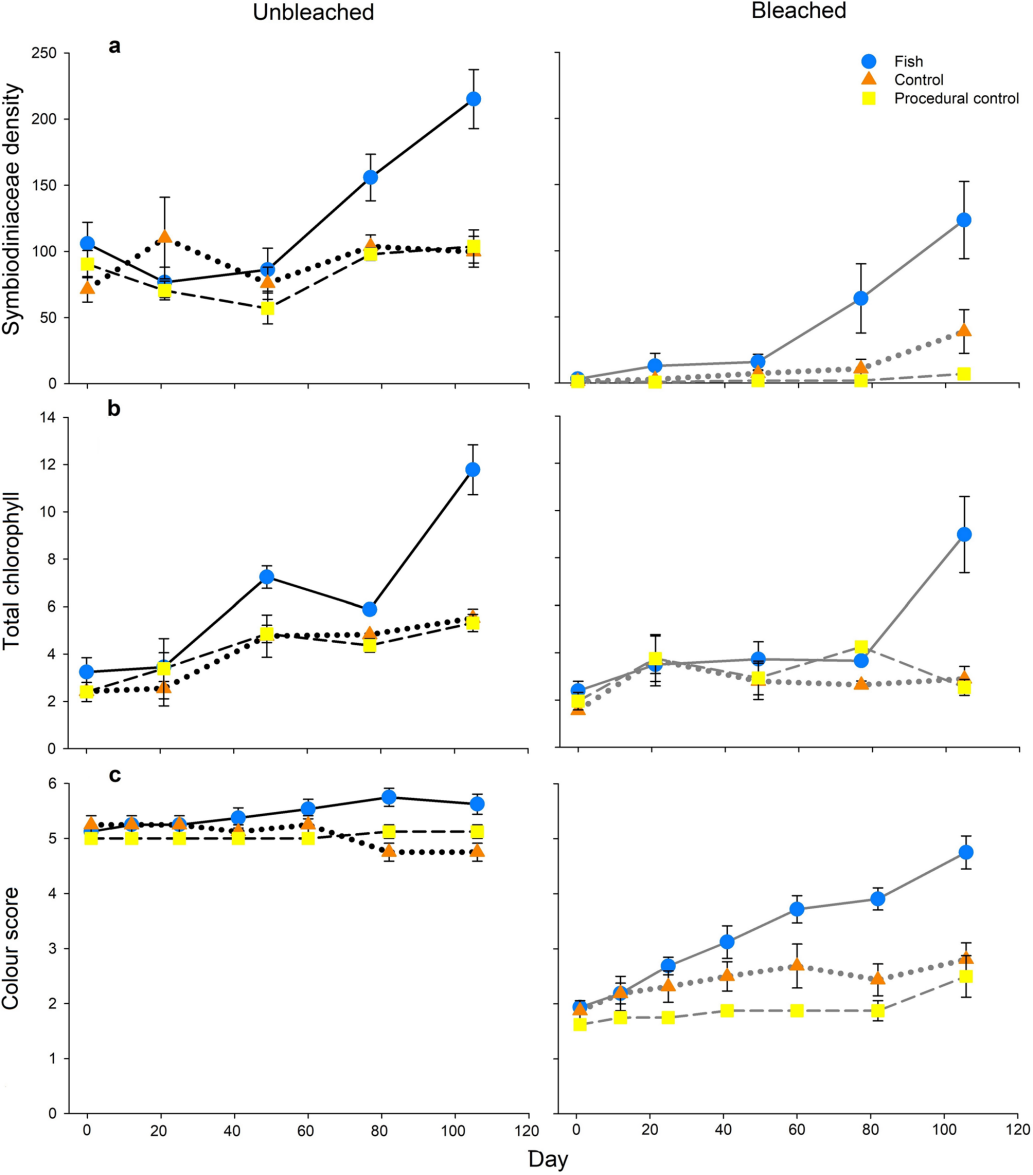
(**a**) Symbiodiniaceae density per mg of host protein, (**b**) total chlorophyll per mg of host protein and (**c**) colour score throughout the experiment (mean ± SE, n = 8). Note the control and procedural control treatments are shown on these graphs. However, these were pooled for statistical analyses and described as such in the supporting text.

At the start of the experiment, there was a clear difference in Symbiodiniaceae density per mg of host protein between the bleached and unbleached sea anemones (Fig. 1a). As time progressed, little change was seen in the bleached and unbleached controls (the bleached procedural control and control are herein pooled, as are the unbleached procedural control, for all tests due to no statistical differences, n = 16 for both, see Supplementary Table 1). However, Symbiodiniaceae densities increased substantially from day 77 in both the bleached and unbleached sea anemones that hosted anemonefish (n = 8 per treatment). After 106 days, Symbiodiniaceae density had fully recovered in the bleached sea anemones with anemonefish, with no significant difference found when tested against the unbleached controls (P = 0.877). In contrast, the bleached controls did not recover: these contained 78% fewer algal symbionts than in the unbleached controls (P = 0.002). Unbleached sea anemones that hosted anemonefish had the highest Symbiodiniaceae density compared to other experimental treatments, increasing 103% over the 106 days (unbleached with anemonefish v bleached with anemonefish, P = 0.021; unbleached with anemonefish v unbleached controls, P < 0.001).

### Total chlorophyll

Total chlorophyll per mg of host protein was similar among all treatments on day 0 (Fig. 1b). Over time, little change was observed in the bleached controls; whereas an increase was seen in all other treatments, with the greatest rate of change occurring in the sea anemones that hosted anemonefish. Both the bleached and unbleached sea anemones with anemonefish had significantly higher chlorophyll than the unbleached controls at the end of the experiment (66% higher, P = 0.005; 117% higher, P < 0.001, respectively). In contrast, total chlorophyll in the unbleached and bleached sea anemones without anemonefish did not differ at the end of the experiment (P = 0.125), and no differences were found between the unbleached and bleached sea anemones with anemonefish (P = 0.106).

### Colour score

**Figure 2:**
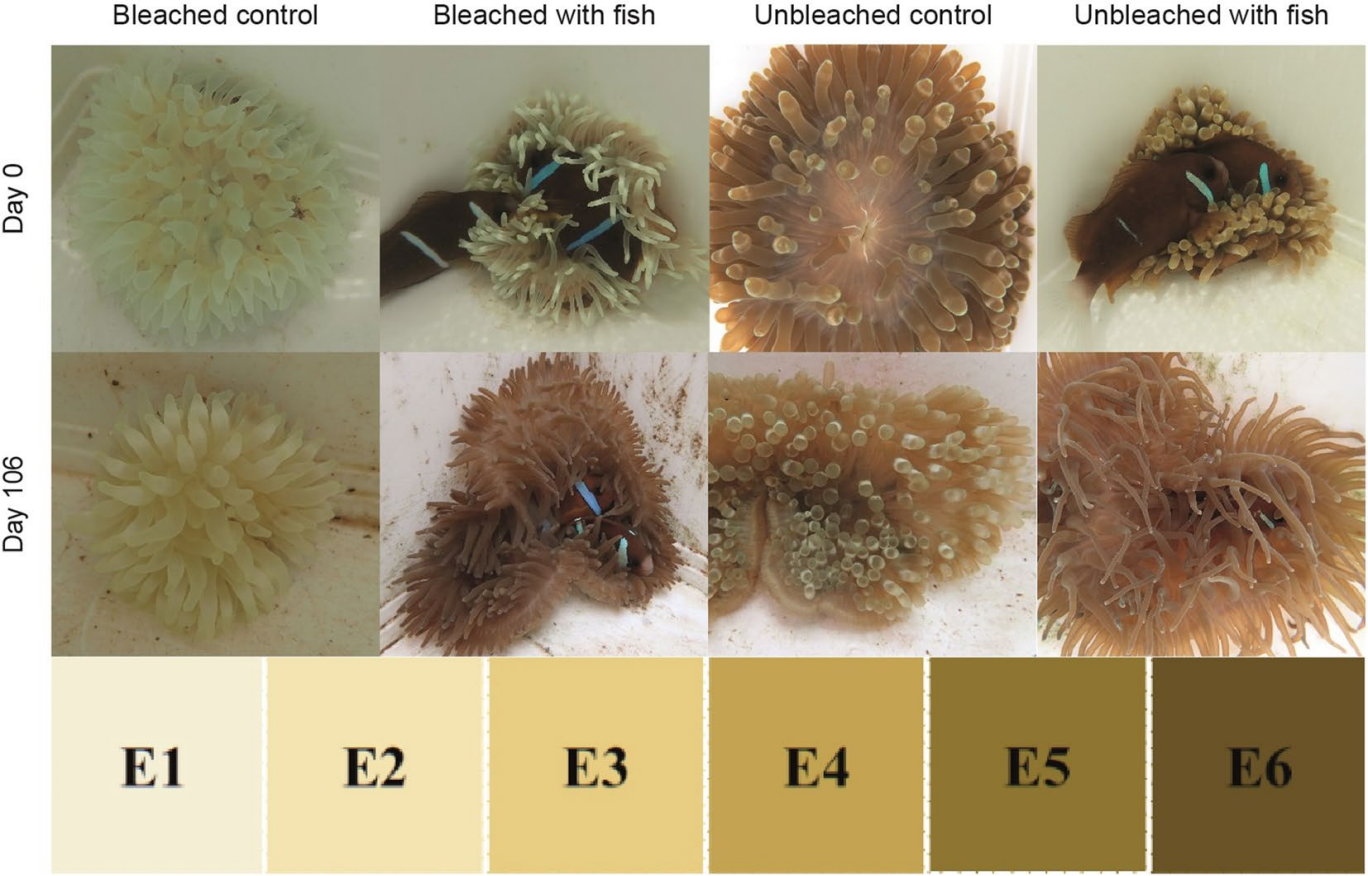
Sea anemones indicative of each treatment at the beginning (day 0) and end (day 106) of the experiment. The E hue colour score from the CoralWatch Coral Health Chart was used to assess visual changes.

Over time, colour score increased only in the sea anemones that hosted anemonefish (Figs. 1c, 2). The colour score of bleached sea anemones with anemonefish was not significantly different from the unbleached controls by day 106 (P = 0.609), indicating recovery. In contrast, the colour score was 46% lower in the bleached controls than unbleached controls (P < 0.001), signifying recovery had not occurred. Overall, the unbleached sea anemones with anemonefish had the highest colour score (unbleached with anemonefish versus bleached with anemonefish, P = 0.033; unbleached with anemonefish versus unbleached controls, P = 0.004).

### Survival

All sea anemones survived, except for one bleached control, and a small fragment (resulting from fission) from one bleached sea anemone with anemonefish. Fission occurred in bleached sea anemones with anemonefish, with a mean of 0.75 ± SE 0.37 clonal descendants per sea anemone by the end of the experiment. For the unbleached sea anemones with anemonefish, there were 0.38 ± 0.26 mean clonal descendants per sea anemone. In contrast, in the bleached sea anemones without anemonefish treatment, there were only 0.06 ± 0.06 mean clonal descendants per sea anemone. Fission did not occur in any of the unbleached sea anemones without anemonefish.

## Discussion

Anemonefish facilitated bleaching recovery in a host sea anemone, thereby enhancing resilience. Both unbleached and bleached sea anemones had increased Symbiodiniaceae density and total chlorophyll when anemonefish were present, which is likely due to the nutrients excreted by the ectosymbiont^17,42^. Sea anemones hosting anemonefish have a distinct ecological advantage that will become increasingly important as sea temperatures continue to rise. Obligate symbioses and reliance on a specialised habitat can impose additional risks to both the habitat and the hosted organism during environmental variation^45,46^. However, here we clearly show how the anemonefish-sea anemone-Symbiodiniaceae association can be beneficial following bleaching.

In this study, Symbiodiniaceae density and total chlorophyll increased considerably for both unbleached and bleached *E. quadricolor* hosting the anemonefish *A. akindynos*. Similarly, for unbleached *E. quadricolor*, Symbiodiniaceae density increases when hosting *A. bicinctus* in the laboratory, and the sea anemone *Heteractis magnifica* grows faster when *A. chrysopterus* are present in the field^17, 40,47^. Additionally, we have shown that anemonefish facilitate bleaching resilience, as Symbiodiniaceae density and colour score showed full recovery in sea anemones hosting anemonefish (i.e. bleached with fish were statistically similar to the unbleached controls). Damselfish have been found to assist coral bleaching recovery by enhancing endosymbiont density and chlorophyll concentration^6^, as has heterotrophic feeding^48–50^. The facilitated recovery, both in this study and by Chase et al.^6^ may be enabled by fishes excreting waste products that are absorbed by the sea anemone and subsequently, their Symbiodiniaceae. The additional nutrients are likely to have increased endosymbiont asexual reproduction, leading to higher density^13,17^. Additionally, ad hoc observations showed that bleached sea anemones spent more time with their tentacles expanded when hosting anemonefish, which would enhance light interception and potentially benefit Symbiodiniaceae, facilitating recovery^40^. Interestingly, total chlorophyll was similar between the bleached and unbleached sea anemones at the start of the experiment, indicating bleaching occurred due to symbiont loss. The similarity in total chlorophyll among treatments is likely due to reduced intraspecific competition between the Symbiodiniaceae in the bleached sea anemones, which may have resulted in higher chlorophyll concentration per cell.

Here, bleaching recovery was dependent on the presence of anemonefish, with Symbiodiniaceae density in fish-hosting anemones being similar to that of the unbleached controls within 2 months, and colour score within 3.5 months. The total chlorophyll was considerably higher in the sea anemones occupied by fish by the end of our experiment. Although horizontal endosymbiont repopulation was not possible due to filtered and UV sterilised seawater used in this experiment, our findings are comparable to the rates observed for *H. magnifica* hosting *A. chrysopterus* in Moorea, French Polynesia^38^. In contrast, only partial recovery was recorded 2–4 months following bleaching of occupied *Stichodactyla haddoni* and *H. crispa* in Bootless Bay, Papua New Guinea, and *E. quadricolor* remained bleached after 6 months at Lizard Island, Australia, despite hosting anemonefish^32, 36^. These findings suggest bleaching recovery in sea anemones is likely to be species-specific, as well as influenced by local environmental conditions and biological factors both during and after bleaching events. In our experiment, nutrients concentrations may not have been equivalent to field conditions, thus altering recovery times. Additionally, recovery in the bleached sea anemones without anemonefish may have been possible if a longer time frame was provided, given Symbiodiniaceae density did slightly increase over 3.5 months.

We found *E. quadricolor* with and without anemonefish survived for extended periods in the laboratory following bleaching. While this was somewhat unexpected, survival is likely to be linked to bleaching severity and environmental variables such as water flow, light levels, and organic matter^44,51–53^. Thus, mortality is not constant among locations and events. For example, 5% of occupied *S. haddoni* and *H. crispa* at Bootless Bay died following three weeks of warming^36^, in comparison to 88% of occupied *H. crispa* at Sesoko Island, Japan, following 13 weeks of elevated temperatures during the 1998 mass bleaching event^34^.

For sea anemones with and without anemonefish, asexual reproduction via longitudinal fission appeared to be influenced by temperature. Previous studies have shown increased fission results from experimentally elevated temperatures in a range of non-host sea anemones^54–57^. In our experiment, fission occurred much more often in sea anemones with anemonefish, regardless of thermal exposure and bleaching status. Therefore, while temperature may have influenced fission, the presence of fish was the overriding causative factor. Similarly, increased fission has been documented in the field for unbleached *H. magnifica* with anemonefish^58^. Fish may accelerate fission by augmenting nutrition, as feeding has been shown to increase asexual reproduction^54,59–61^.

By elucidating a previously unknown benefit of the anemonefish-sea anemone-Symbiodiniaceae symbiosis, our findings add to the growing body of literature that shows interactive relationships can enhance resilience to climate change^4–8^. We found that bleached sea anemones with anemonefish were able to recover, whereas those without anemonefish did not. Given severe bleaching events are increasing in frequency^10,62^, and can result in decreased sea anemone and anemonefish abundance^31–34^, anemonefishes are likely to enhance host sea anemone survival as our oceans continue to warm. Symbioses between fishes, such as gobies and damselfish, and cnidarians that bleach are common on coral reefs^63,64^, and can also provide resilience to bleaching^6^. Therefore, while this study used the anemonefish-sea anemone symbiosis, the findings are not uniquely relevant to this model system. These results highlight the need to ensure symbioses are maintained and reinforce the importance of management and conservation efforts focusing on ecosystem, rather than single-species, approaches.

## Methods

### Collection and acclimation of *Entacmaea quadricolor* and *Amphiprion akindynos*

*Entacmaea quadricolor* (N = 48) that did not host anemonefish were collected from 13-m depth in July 2017 (austral winter) at North Solitary Island, Australia (29°55′54ʺ S, 153°23′21ʺ N). Unoccupied sea anemones were used to ensure the presence of anemonefish did not impact Symbiodiniaceae density before experimentation^17^, and to minimise potential adverse impacts to the anemonefish population at the collection location. As colour morph has been shown to influence bleaching susceptibility for this species^44,65^, all sea anemones had a green column, brown tentacles with green and white pigmentation at the tips and a brown oral disc. After collection, individuals were transported to the National Marine Science Centre (NMSC), Coffs Harbour, New South Wales, Australia, and kept in a 2000 L rectangular outdoor tank with ambient flow-through seawater (20.5 °C, reflecting temperature at the collection location, 8 L min^−1^) for 26 days. Light levels were reduced by 70% shade cloth to simulate conditions at the collection location. Water temperature within the outdoor tank was increased from 20.5 to 22 °C by ramping 0.5 °C every 2 days using thermostatically controlled heater-chiller units, reflecting summer temperatures experienced in the collection region. Sea anemones were kept at this temperature for 53 days and fed *Melicertus plebejus *(Eastern King Prawn, ~ 1 cm^3^) flesh fortnightly.

*Amphiprion akindynos* form a symbiotic relationship with *E. quadricolor.* These fish were collected as pairs consisting of one female and one male (N = 16 pairs, total length = 10.3 ± SE 0.18 cm) from North Solitary Island*.* Pairs were then transported to the NMSC and held at ambient temperature (20.5 °C) with flow-through seawater (200 mL min^−1^) in individual 42 L white plastic aquaria (432 mm long × 324 mm wide × 305 mm deep). Before the experiment commenced, terracotta pots were provided as habitat and fish were fed pellets (Hakari Marine A) twice daily. Fish were collected under a NSW Department of Primary Industries Scientific Collection Permit (P17/0042–1.1). Experimental protocols were carried out in accordance with NSW Animal Research Act (1985) and Regulation (2010), and approved by Southern Cross University Animal Care and Ethics Committee (Animal Research Authority 17/42).

### Sea anemone bleaching

Sea anemones were divided equally between two 2,000 L tanks, and the temperature was increased to 27 °C over 24 h (0.5 °C.2 h^−1^) in one tank to induce thermal bleaching, whereas the other was maintained at 22 °C. The higher temperature aligns with future ocean temperature predictions^66^, in addition to bleaching thresholds found for *E. quadricolor* in this region^44^. These conditions were maintained for 48 days, during which the sea anemones were monitored with a CoralWatch Coral Health Chart to assess bleaching^67,68^. This chart uses a brightness/saturation score, with four colour hues (here, the E hue was used), each with a score ranging from one to six, with a two-point reduction indicating a significant loss of Symbiodiniaceae and chlorophyll *a*^68^. Colour score was determined halfway down the tentacle where host pigmentation was least likely to obscure the reading, and therefore the score would be more indicative of bleaching. After exposure to increased temperatures, Symbiodiniaceae density was 17,816 ± SE 3,995 per mg of host protein, and colour score was 1.8 ± 0.07 for the bleached sea anemones, in comparison to 672,141 ± 84,168 symbionts per mg of host protein and colour score of 5.1 ± 0.07 , for those maintained at 22 °C (i.e. unbleached). Therefore, Symbiodiniaceae remained present in all sea anemones, making vertical repopulation possible. While all sea anemones were a similar size before bleaching (tentacle crown diameter of 13.3 ± 0.3 cm), variation was apparent after thermal exposure (tentacle crown diameter in unbleached sea anemones was 14.9 ± 1.0 cm, and for the bleached sea anemones was 9.3 ± 0.5 cm).

### Experimental conditions

Sea anemones were randomly placed into individual 42 L white plastic aquaria (432 mm long × 324 mm wide × 305 mm deep), divided evenly among four 2000 L water tables (2000 mm long × 1000 mm wide × 1000 mm deep). Initially, heated seawater was supplied to bleached sea anemones (27 °C, n = 24), and then gradually decreased to 23 °C over 24 h. Once the average summer temperature of 23 °C was reached, the experiment began, and therefore time 0 was 24 h post thermal bleaching. To reduce the likelihood of pathogens entering the experimental system and adversely affecting the anemonefish, filtered (50 µm) and UV sterilised (50 W) seawater was supplied (200 mL min^−1^) to all aquaria. Temperature and light intensity were recorded every 15 min, using 16 and 3 randomly placed loggers, respectively. Shade cloth was placed over the tanks to achieve light levels similar to field conditions. The maximum light intensity reached was 695.5 μmol photons m^−2^ s^−1^ (see Supplementary Information Fig. 1).

### Treatments

Sea anemones were randomly assigned to one of six treatments, each with eight replicates: (i) bleached with an anemonefish pair; (ii) unbleached with an anemonefish pair (one female, one male); (iii) bleached without anemonefish (bleached control); (iv) unbleached without anemonefish (unbleached control); (v) bleached without anemonefish but with fish food pellets added (bleached procedural control) and; (vi) unbleached without anemonefish but with fish food pellets added (unbleached procedural control). *Amphiprion akindynos* were fed pellets (Hakari Marine A) to satiety twice daily. Food was added at the opposite end of the aquaria to the sea anemone, and uneaten pellets were removed after 30 min. The procedural control also had inaccessible fish food pellets placed in the same location for an equal duration.

Food was also provided directly to the sea anemones to account for heterotrophic feeding in the field. Little information is available on the frequency or amount of feeding that would naturally occur for *E. quadricolor*. Therefore, a conservative approach was taken, with all sea anemones being fed *M. plebejus *flesh (~ 1 cm^3^) every four weeks.

### Tentacle removal and processing

Tentacles were collected on days 0, 21, 49, 77 and 106 to determine host protein, Symbiodiniaceae density, and total chlorophyll concentration. If a sea anemone had split, samples were taken from each fragment and averaged. For each sea anemone, 3–4 tentacles were removed using dissection scissors and placed in a 10 mL centrifuge tube containing 4 mL of 0.4 μm filtered seawater. Samples were homogenised at 15,000 rpm. The homogenate was centrifuged at 4500 rpm for 15 min until the Symbiodiniaceae pellet separated from the sea anemone supernatant.

### Host protein analysis

Host supernatant was removed, placed into 10 mL digestion vials and frozen at − 20 °C, then later analysed to determine protein content. The samples were transported on ice to the Environmental Analysis Laboratory, Southern Cross University, Australia. Once thawed, samples were diluted 100 times with ultrapure water, acidified with nitric acid digestion solution, and filtered with a 0.45 μm cellulose acetate filter. Nitrogen content (mg. L^−1^) was determined using Inductively Coupled Plasma Mass Spectrophotometry^69^, and converted to protein content by multiplying by 6.25^70^.

### Symbiodiniaceae density

For each sample, the Symbiodiniaceae pellet was re-suspended in 4 mL of 0.4 μm filtered seawater and homogenised for 10 s at 15,000 rpm. Homogenate (1 mL) was placed into 1.5 mL microcentrifuge tubes to determine Symbiodiniaceae density. Using a haemocytometer, either 8 or 12 replicate counts were done per sample with either 5 or 25 fields per count, depending on cell density. More replicates and fields were used for low density (≤ 10 cells per field) counts to ensure accuracy. Counts were standardised against host protein.

### Chlorophyll concentration

To determine total chlorophyll (chlorophyll *a* + *c*_2_), the remaining 3 mL of solution was re-centrifuged at 4500 rpm for 15 min, and the supernatant was discarded. The algal pellet was re-suspended in 4 mL of 90% acetone and kept for 24 h at 4 °C in the dark to avoid photodecomposition while exposed to this solvent. The suspension was centrifuged once more at 4500 rpm for 15 min. The supernatant was then placed into a cuvette and absorbance at 630, and 664 nm was measured on a Bibby Anadéo spectrophotometer. Total chlorophyll concentration was determined using the equations of Jeffrey and Humphrey^71^ and expressed in mg of host protein.

### Visual observations

Colour score was recorded on days 1, 12, 25, 41, 60, 82 and 106 using a CoralWatch Coral Health Chart^67,68^. Three tentacles from each sea anemone were observed, and an average calculated for each individual. Sea anemones were monitored visually twice daily for survivorship and asexual reproduction via longitudinal fission.

### Statistical analysis

Permutational Multivariate Analysis of Variance (PERMANOVA) in PRIMER v6 + PERMANOVA was conducted to determine statistical differences among treatments. Univariate resemblance matrices were generated using Euclidean Distance. Permutational Analysis of Multivariate Dispersion (PERMDISP) was used to test for significant deviation from the centroid, and if found, data were log (x + 1) transformed. As no significant effect was found with the nested tank factor, a one-way design was used comparing treatments. Within this design, a priori comparisons were set up to test for differences between the bleached control and bleached procedural control, and between the unbleached control and unbleached procedural control. For these tests, no statistically significant differences for Symbiodiniaceae density, total chlorophyll or colour score were found, and so the bleached control and bleached procedural control treatments were pooled, as were the unbleached control and unbleached procedural control treatments. Further comparisons of Symbiodiniaceae density, total chlorophyll and colour score were made to test the following hypotheses: values in bleached sea anemones with anemonefish were significantly greater than bleached controls (pooled); values in bleached sea anemones with anemonefish did not differ significantly from unbleached controls (pooled); values in unbleached sea anemones with anemonefish were significantly greater than bleached with anemonefish and; values in unbleached sea anemones with anemonefish were significantly greater than unbleached controls (pooled). All PERMANOVAs were tested using 4999 raw data permutations with Type III sum-of-squares. Outputs from the statistical analyses can be found in the results section of this manuscript and Supplementary Information Table 1.

## Supplementary information

Supplementary Information

## Data Availability

Data are available at https://doi.org/10.5281/zenodo.3551987.
