# Supplementary material for: Anemonefish facilitate bleaching recovery in a host sea anemone
Source: Sci Rep. 2020 Oct 29;10:18586. doi: 10.1038/s41598-020-75585-6 (PMC7596230; doi:10.1038/s41598-020-75585-6)
Supplement: Supplementary file 1 — Supplementary Information [file 41598_2020_75585_MOESM1_ESM.pdf]

Anemonefish facilitate bleaching recovery in a host sea anemone

Sophie H Pryor[1], Ross Hill[2], Danielle L Dixson[3], Nicola J Fraser[1], Brendan P Kelaher[1], Anna Scott[1*]

[1]National Marine Science Centre, Marine Ecology Research Centre, School of Environment, Science and Engineering, Southern Cross University, P.O. Box 4321, Coffs Harbour, NSW, 2450, Australia

[2]Macquarie University, Sydney, NSW, 2109, Australia

[3]School of Marine Science and Policy, University of Delaware, Lewes, DE 19958, USA

*Corresponding author email: anna.scott@scu.edu.au

Supporting information

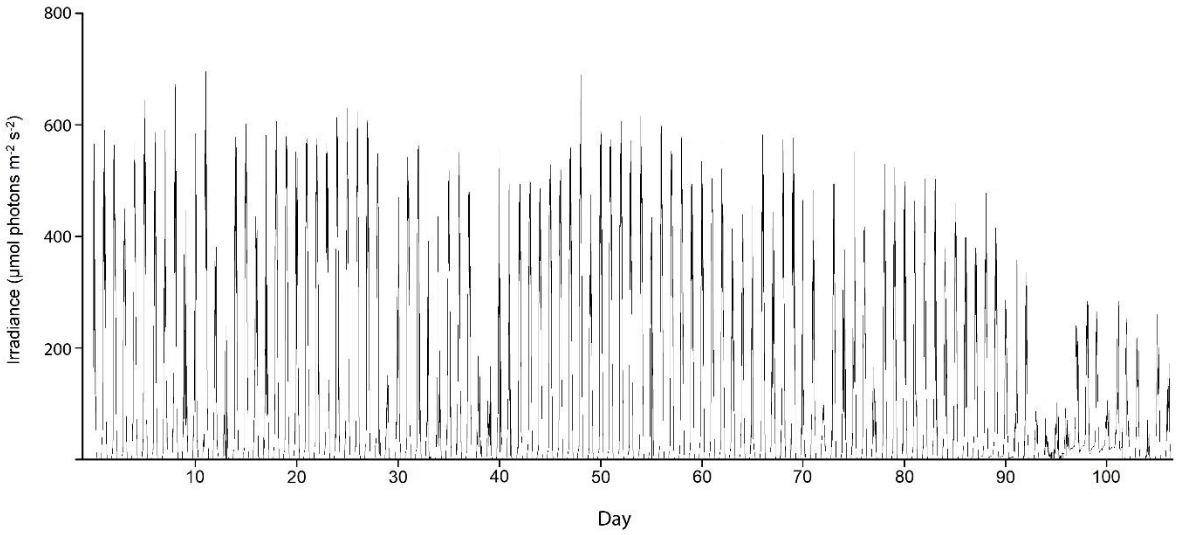

Supporting Figure 1: Irradiance (μmol photons m^{-2} s^{-2}) during the bleaching recovery period.

Supporting Table 1: P-values calculated from PERMANOVA to test hypotheses. Asterisks (*) indicate where the null hypothesis was rejected. ± = SE.

Hypothesis	Symbiodiniaceae density	Total chlorophyll mg host protein^{-1}	Colour score
Treatment effect	P = 0.003*	P < 0.001*	P < 0.001*
Bleached control = bleached procedural control	0.2888	0.4178	0.9214
Unbleached control = Unbleached procedural control	0.2694	0.9708	0.7200
Bleached with fish = unbleached controls (pooled)	P = 0.877* Means = 1232242 ± 291741.0, 1017974 ± 120879.8	P = 0.005 Means = 8.98 ± 1.610, 5.42 ± 0.373	P = 0.609* Means = 4.75 ± 0.299, 4.94 ± 0.144

Bleached controls (pooled) < unbleached controls (pooled)	P = 0.002* Means = 228801 ± 98744.2, 1017974 ± 120879.8	P = 0.125 Means = 2.71 ± 0.434, 5.42 ± 0.373	P < 0.001* Means = 2.66 ± 0.338, 4.94 ± 0.144
Unbleached with fish > Bleached with fish	P = 0.021* Means = 2152018 ± 223212.0, 1232242 ± 291741.0	P= 0.106 Means = 11.78 ± 1.057, 8.98 ± 1.610	P = 0.033 Means = 5.63 ± 0.183, 4.75 ± 0.299
Unbleached with fish > Unbleached controls (pooled)	P < 0.001* Means = 2152018 ± 223212.0, 1017974 ± 120879.8	P < 0.001* Means = 11.78 ± 1.057, 5.42 ± 0.373	P = 0.004* Means = 5.63 ± 0.183, 4.94 ± 0.144
